# Development of Next Generation *Streptococcus pneumoniae* Vaccines Conferring Broad Protection

**DOI:** 10.3390/vaccines8010132

**Published:** 2020-03-17

**Authors:** Malihe Masomian, Zuleeza Ahmad, Lai Ti Gew, Chit Laa Poh

**Affiliations:** 1 Centre for Virus and Vaccine Research, School of Science and Technology, Sunway University, Kuala Lumpur, Selangor 47500, Malaysia; malihem@sunway.edu.my (M.M.);; 2 Department of Biological Sciences, School of Science and Technology, Sunway University, Kuala Lumpur, Selangor 47500, Malaysia

**Keywords:** *Streptococcus pneumoniae*, vaccines, live attenuated vaccine, pneumococcal surface-exposed protein, nanoparticle, bacterium-like particle

## Abstract

*Streptococcus pneumoniae* is a major pathogen causing pneumonia with over 2 million deaths annually, especially in young children and the elderly. To date, at least 98 different pneumococcal capsular serotypes have been identified. Currently, the vaccines for prevention of *S. pneumoniae* infections are the 23-valent pneumococcal polysaccharide-based vaccine (PPV23) and the pneumococcal conjugate vaccines (PCV10 and PCV13). These vaccines only cover some pneumococcal serotypes and are unable to protect against non-vaccine serotypes and unencapsulated *S. pneumoniae*. This has led to a rapid increase in antibiotic-resistant non-vaccine serotypes. Hence, there is an urgent need to develop new, effective, and affordable pneumococcal vaccines, which could cover a wide range of serotypes. This review discusses the new approaches to develop effective vaccines with broad serotype coverage as well as recent development of promising pneumococcal vaccines in clinical trials. New vaccine candidates are the inactivated whole-cell vaccine strain (Δ*pep27*Δ*comD* mutant) constructed by mutations of specific genes and several protein-based *S. pneumoniae* vaccines using conserved pneumococcal antigens, such as lipoprotein and surface-exposed protein (PspA). Among the vaccines in Phase 3 clinical trials are the pneumococcal conjugate vaccines, PCV-15 (V114) and 20vPnC. The inactivated whole-cell and several protein-based vaccines are either in Phase 1 or 2 trials. Furthermore, the recent progress of nanoparticles that play important roles as delivery systems and adjuvants to improve the performance, as well as the immunogenicity of the nanovaccines, are reviewed.

## 1. Introduction

*Streptococcus pneumoniae* or pneumococcus is a common human pathogen contributing to significant morbidity and mortality annually, especially in children, the elderly, and the immunocompromised [1]. *S. pneumoniae* remains the leading cause of community-acquired pneumonia (CAP) despite the worldwide administration of pneumococcal vaccines [2]. *S. pneumoniae* can also cause a myriad of non-invasive and invasive diseases. Non-invasive pneumococcal diseases include sinusitis, acute otitis media, and pneumonia that is localized to the lungs [3,4]. The invasive form of pneumococcal pneumonia can lead to bacteremia and meningitis [3]. In the US, pneumococcal sepsis and meningitis contributed to a few thousand deaths annually in adults. The pneumococcus normally colonizes the nasopharynx asymptomatically and colonized humans serve as an effective reservoir for the pneumococcus, facilitating the transmission of the bacteria in the community [3,5]. There are at least 98 serotypes of pneumococcus circulating worldwide, categorized according to the unique glycan components and linkages that constitute the capsular polysaccharide of each serotype [6]. However, the 10 most common types cause 62% of invasive disease worldwide [7].

## 2. Current Pneumococcal Vaccines in the Market

There are two types of pneumococcal vaccines that are available in the market: Pneumovax23 or 23-valent pneumococcal polysaccharide-based vaccine (PPV23) and Pneumococcal conjugate vaccines (PCVs). Pneumovax23 (PPV23) was licensed in 1983 and is distributed by Merck (Lansdale, PA, USA). This polysaccharide vaccine was manufactured by purifying the capsular polysaccharide antigens from 23 different serotypes of the pneumococcus [8]. These 23 serotypes are responsible for 85–90% of invasive pneumococcal infections in the world. PPV23 is recommended for individuals aged 65 years and above as well as individuals aged 2 to 64 who had comorbidity, such as chronic cardiovascular disease and diabetes [9]. The effectiveness of PPV23 appeared to be dependent on whether the measured outcome is due to the incidence of invasive or non-invasive pneumococcal diseases. Studies showed the vaccine could only lessen the severity of CAP but not prevent it, and it could not reduce the incidence of non-invasive pneumonia and morbidity [10,11]. This is likely due to PPV23, which could only elicit serum IgG but not secretory IgA in the nasopharynx [12]. However, PPV23 is thought to be effective in preventing invasive pneumococcal disease (IPD) in healthy individuals under 75 years [13]. It is widely accepted that PPV23 is not effective in children due to the inability of the vaccine to generate immunological memory [14,15] and the vaccine also did not lead to reduced carriage [16].

Pneumococcal conjugate vaccines (PCVs) were first introduced in 2000 in the form of PCV7. The current PCVs in the market are Prevnar13, manufactured by Pfizer, and a relatively newer 10-valent pneumococcal non-typeable *Haemophilus influenza* protein D conjugate vaccine (PHiD-CV), manufactured by GlaxoSmithKline plc. (Brentford, UK) [17]. PCV13 was formulated by conjugating capsular polysaccharide antigens with the diphtheria toxoid carrier protein, CRM_197_ [18]. PHiD-CV contained pneumococcal polysaccharides of eight serotypes conjugated to the non-typeable *Haemophilus influenza* carrier protein D, serotype 18C conjugated to tetanus toxoid, and serotype 19F conjugated to diphtheria toxoid, leading to a 10-valent vaccine [19]. Both vaccines include the serotypes causing the majority of IPD in the world, including serotype 19A, which is the most common IPD-causing serotype in young children [20,21]. PCV vaccines were able to confer better immunogenicity due to their ability to elicit memory T cell response [22]. Therefore, younger infants are the target group for PCVs with a dose that is advised to be given 2p + 1 (two primary doses before 6 months of age and one booster dose at 9 months of age) or a 3p + 0 (three primary doses before 9 months of age without a booster dose) schedule [23]. PCVs were reported to reduce pneumonia in children [24] and the vaccine did not interfere with the immune responses to co-administer routine pediatric vaccines [25]. Herd immunity was also achieved due to the ability of the PCVs to reduce carriage [26], which subsequently lowered vaccine-type IPD and non-IPD cases in both vaccinated and unvaccinated individuals [27,28]. It is worth noting that the vaccine effectiveness is contingent on the serotypes included in the vaccine as the overall vaccine effectiveness of PCV13 was 33.2% {95% Confidence Interval(CI)—106.6% to 82%} against pneumococcal CAP irrespective of serotype and 38.1% (95% CI—131.9% to 89%) against vaccine-type-CAP in the cohort of adults ≥ 65 years [29]. Unfortunately, the number of serotypes that could be included in a PCV formulation could not be increased easily as the production of PCV is complex and expensive [30].

The introduction of pneumococcal vaccines has increased the prevalence of pneumococcal disease caused by the serotypes not included in the vaccine formulation [31]. This is referred to as serotype replacement due to the greater diversity of serotypes in the developing countries. To date, there are at least 98 serotypes of pneumococcus that have been identified [32,33]. With the implementation of pneumococcal vaccines, the niches vacated by the vaccine serotypes tend to be colonized by non-vaccine serotypes [34]. In a study of Alaska native children, invasive pneumococcal disease (IPD) rates were found to have returned to pre-vaccine levels due to non-vaccine serotypes [35]. There is a tendency for the non-vaccine serotypes to be highly invasive and possessing higher levels of antibiotic resistance [36,37]. PHiD-CV vaccination was associated with a delayed, relatively lower increase in the non-vaccine-type carriage in clinical studies. However, the problem of serotype replacement still existed and was not eliminated with the administration of PHiD-CV [38]. In addition, geographical differences in serotype distribution have led to reduced effectiveness of the vaccines when they were implemented in geographic areas where the serotypes were not covered by the vaccines [21].

The propensity of *S. pneumoniae* to acquire new genetic materials via natural transformation and recombination makes it one of the deadly human pathogens. This enables the pneumococcus to evolve according to the host environment [39]. Pneumococcus is known to undergo frequent recombination [40,41]. Capsule switching was reported to occur more often following the introduction of pneumococcal vaccines [42]. Moreover, mutations that disrupted the loci, which encoded for capsular polysaccharide biosynthesis, could lead to the emergence of unencapsulated, non-typeable pneumococcus [43]. The pneumococcal surface protein K in these bacterial variants has been suggested to assist in adhesion, contributing to 3–19% of pneumococcal diseases [44]. Acute otitis media caused by non-invasive and non-fatal pneumococcus does exert a large economic burden as it is one of the most common disorders requiring medical care for children [45,46]. Unfortunately, the prevention of acute otitis media caused by serotypes included in PCV remained moderate to low [47,48]. PHiD-CV was shown to be more effective in preventing acute otitis media than PCV7 [38,49].

## 3. Novel Strategies for the Development of New Vaccines against *Streptococcus pneumoniae*

Severe diseases caused by pneumococcus still occur despite the availability of the pneumococcal polysaccharide (PPV23) and conjugate (PCV13) vaccines in the market. The fast spread of multidrug-resistant strains has accelerated the global problem of pneumococcal diseases. The limitations posed by the current PCV vaccines include high costs and insufficient serotype protection [12,50]. Although the introduction of PPVs and PCVs have effectively reduced the disease burden, emerging new clinical isolates require adding newer serotypes to vaccine formulations [51,52,53]. Considering that there are at least 98 serotypes of pneumococcus, it would be more complex and expensive to increase the number of vaccine serotypes in the PCV vaccine formulations. Hence, there is a need to develop new pneumococcal vaccines. A pneumococcal vaccine that exhibits the potential to provide broad serotype coverage, induce mucosal and systemic immunity, and hinder primary intranasal colonization, as well as invasive disease, is much desired. Several pneumococcal proteins have been studied as vaccine candidates such as the pneumococcal surface protein A (PspA) pneumolysin (Ply), pneumococcal surface protein C (PspC), and pneumococcal surface adhesin A (PsaA), which have been comprehensively reviewed by many researchers [54,55,56,57]. Here, we focus on highlighting new approaches to developing the whole-cell vaccines, discovering new protein antigens or genetic manipulations of protein antigens to make more effective vaccines for the next generation *S. pneumoniae* vaccines.

### 3.1. Protein-Based Vaccines

Pneumococcal virulence proteins that have well-conserved sequences and could confer broad serotype coverage are desirable. They are less expensive than PCVs to manufacture. In the past, protein vaccine developments were based on the antigens with known functions in bacterial pathogens, but reverse vaccinology and ANTIGENome technology have contributed to the discovery of novel protein antigens [58,59]. The highly conserved proteins with broad serotype coverage have been extensively investigated as vaccine candidates are listed in Table 1.

Pneumococcal surface protein A (PspA) is a choline-binding protein present on the cell surface of almost all pneumococcal strains. The N-terminal domain of PspA is exposed on the surface and the sequence variations are used to classify pneumococcal strains into three families and six clades. Almost 98% of clinical isolates are from families 1 (clades 1 and 2) and 2 (clades 3 to 5) [60]. PspA is known to inhibit the complement-mediated clearance of pneumococci. The PspA vaccine was shown to be cross-protective in animal models against multiple serotypes causing carriage and invasive diseases [61,62,69]. Phase I clinical trials demonstrated the safety and ability to elicit strong antibody responses in the murine model [70]. However, there is concern to use the whole PspA as it has homology to the human cardiac myosin and could elicit autoantibodies that might promote cardiac inflammation or autoimmune disease [71]. Therefore, novel strategies to develop the PspA-based vaccine would focus on regions of the PspA that do not contain any homology with myosin.

The N-terminal domain of PspA has an α-helical coiled-coil structure and is divided into three regions, A, B and C. The B region exhibits serological variability and is known as a clade-defining region [72]. Akbari et al. constructed a recombinant PspA-based protein vaccine (PspAB_1-5_) comprising the B region fragments from clades 1 to 5 of both families 1 and 2. In the mice immunized with PspAB_1-5_, an increased in C3 complement component deposition on the pneumococcal surface was found. The anti- PspAB_1-5_ antibodies showed cross-reactivity against pneumococci from clades 1, 2, and 5. In addition, the PspAB_1-5_ vaccine was able to confer protection in immunized mice against a *S. pneumoniae* strain from clade 2 in a challenge study. Therefore, the PspA-based vaccine derived from the B region of all clades would be able to confer significant protection against multiple serotypes of *S. pneumoniae* and could serve as key antigenic epitopes to overcome the limitations of the polysaccharide and conjugated vaccines [73].

Besides the N-terminal α-helical domain, which is responsible for eliciting cross-protective immunity, the proline-rich domain (PRD) in the center (~90 amino acids) of the PspA was able to elicit protective antibodies against pneumococcal infection [74]. The diversity of the PRD sequence from 123 pneumococcal isolates led to the recognition of the three PRD groups. Sera from 12 healthy human adults showed antibodies to PRD fragments from pneumococcal strains belonging to each of the three PRD groups. This suggested that a PspA-based vaccine should include conserved epitopes from the PRD of each of the three groups and the α-helical highly charged domain. This approach could provide broad coverage and reduced the likelihood of PspA variants to escape immunity [75].

Pneumococcal surface protein C (PspC) is a cell-wall associated surface protein that is a major virulence factor in almost all pneumococci [76]. PspC plays an important role in bacterial adhesion, invasion, and evasion of complement [77]. It functions as an adhesin by interacting with the secretory component of immunoglobulin A (SIgA) and laminin-integrin receptors [78]. The ability of PspC to bind directly with complement component C3 and recruit factor H to the bacteria surface led to reduction in complement activation. Several studies have demonstrated that PspC is highly immunogenic and the anti-PspC antibodies were able to confer protection against carriage and challenge against invasive disease in mice. Despite the presence of the *PspC* gene in almost all pneumococci, it is highly polymorphic and PspC variants were found to differ in their capacity to bind factor H. It was reported that a *S. pneumoniae* strain, which expressed a PspC variant matching the antibody specificity was killed efficiently, but antibody was not effective against mismatched PspC variants [79]. Due to the high diversity in the protein sequence of the PspC, the whole protein might not serve as an ideal vaccine for cross-protective immunity.

Pneumolysin (Ply) is a cytotoxic cholesterol-binding protein, which is released during autolysis of bacteria that can form pores in eukaryotic membranes [65,66]. Besides its lytic activity, Ply is able to activate complement and the innate immune system through the toll-like receptor-4 (TLR4) and NOD-, LRR- and pyrin domain-containing protein 3 (NLRP3) inflammasome [80,81,82]. Activation of complement by Ply is known to cause complement depletion and reduces complement binding to pneumococcus. However, in non-immune mice and unvaccinated humans, C3 deposition was increased in pneumococcus [83]. Non-toxic variants of Ply were found to produce much less inflammation in the mouse lung [84]. It was reported that TLR4-deficient mice were more easily colonized and showed higher susceptibility to sepsis [81].

The pneumococcal histidine triad proteins (Phts) are a family of surface proteins that have an unusual polyhistidine motif, HXXhXH, which is repeated five or six times in their protein sequences. Pneumococcal histidine triad protein D (PhtD) functions as an adhesin and has a high affinity for zinc [67]. The high levels of zinc in the nasopharynx and lung facilitated the greater surface attachment of pneumococcus. It is a highly conserved surface protein. The PhtD vaccines were able to protect the immunized mice and primates against pneumococcal nasopharyngeal and lung colonization [85,86,87,88]. A recombinant PhtD-based protein vaccine constructed by Seiberling et al. was shown to be safe and immunogenic in adults (18–50 years) in a Phase 1 clinical trial and the second booster was able to increase the levels of anti-PhtD antibodies [89]. The human anti-PhtD antibodies were found to be functional in mice and conferred passive protection [90].

Elongation factor Tu (EF-Tu) is a surface-accessible protein found in the bacterial cytoplasm and culture supernatant. EF-Tu is among the most conserved and universally expressed factors in *S. pneumonia* and other pathogenic bacteria such as *Streptococcus pyogenes*, *Staphylococcus aureus*, and *Pseudomonas aeruginosa*. EF-Tu displays molecular chaperone activity and it is involved in peptide biosynthesis, protein folding and cell response to stress [91]. Nagai et al. showed that *S. pneumoniae* released EF-Tu through autolysis and this was followed by the induction of proinflammatory cytokines in macrophages via the toll-like receptor 4 [68]. A recombinant vaccine was developed containing only the EF-Tu protein of *S. pneumoniae* strain D39 against pneumococcal infection. EF-Tu was produced intracellularly and was presented on the surface of different *S. pneumoniae* strains belonging to serotypes 3, 7F, 10A, 12F, 15A, and 19A. Immunization of mice with recombinant (r) EF-Tu showed significant enhancement in the production of cytokines including IL-6, TNFα, IFN-γ, and IL-17. In addition, an increased CD4^+^ T-cell population in splenocytes as well as IgG1 and IgG2a antibodies were observed in murine models. The anti-EF-Tu serum showed an increase of phagocytic activity of the peritoneal macrophages against *S. pneumoniae*, independent of their serotypes. The mice immunized with recombinant EF-Tu were shown to be protected against lethal challenges with *S. pneumoniae* serotype 2 and the multidrug-resistant serotype 15A [92]. Hence, pneumococcal EF-Tu could be a potential broad-spectrum antigen candidate for a new vaccine against common pneumococcal serotypes.

### 3.2. Whole-Cell Vaccines

Whole-cell vaccines are able to express all protein antigens without requiring purification of individual proteins. Thus, it could serve as an economical alternative to current polysaccharide-based vaccines in developing countries. Several studies showed that the killed whole-cell or live-attenuated vaccines from unencapsulated *S. pneumonia* were able to confer serotype-independent protection, both humoral and cellular responses against multiple antigens in animals [93,94,95,96,97].

#### 3.2.1. Killed Whole-Cell Vaccine

The idea of a live pneumococcal attenuated vaccine posing greater risks is due to the propensity of *S. pneumoniae*. It can lyse the cells using the lytA-dependent mechanism and release the toxin pneumolysin along with its genetic materials that could be taken up by neighboring pneumococcal cells [98]. Therefore, an inactivated vaccine could be much safer than a live attenuated vaccine. Malley et al. constructed a killed whole-cell vaccine (WCV) using the *S. pneumonia* strain RX1, which is a capsule-negative mutant derived from a pneumococcus capsular serotype 2. The strain RX1 was mutated by deleting an autolysin (*lytA*) and killed using ethanol [97]. LytA is a virulence factor involved in autolysis and penicillin-induced lysis [99]. The vaccine was administered intranasally with cholera toxin (CT) as an adjuvant. It was able to confer protection against nasal colonization and invasive disease in immunized mice and rats against encapsulated serotypes 6B and 3 strains in a challenge study [97]. Administration of the vaccine by using nontoxic adjuvants such as cholera toxin binding subunit (CTB) was shown to significantly decrease the nasopharyngeal and middle ear colonizations by serotypes 6B, 14, 23F in murine models [100]. Later, the pneumolysin (*Ply*) gene was also knocked out in strain RX1 and substituted with pneumolysoid. Pneumolysoid is a derivative of *Ply* that contains the toxin gene with 3-point mutations known to eliminate both cytolytic activity and complement activation but maintains its TLR4 agonistic properties. Lu et al. replaced the entire *lytA* gene with the kanamycin resistance gene to improve the yield by reducing the autolysis and named the strain RM200 (RX1E PdT*ΔlytA*) [101]. The beta-propiolactone inactivated RM200 whole-cell vaccine strain (WCV) was shown to confer good protection against nasopharynx colonization against serotype 6B and activated interleukin-17 A (IL-17A) priming [101]. The preclinical study demonstrated that the administration of the beta-propiolactone inactivated WCV with aluminum hydroxide as an adjuvant could significantly induce the IgG titers and IL-17 response [102]. Therefore, the vaccine has been progressed to be evaluated in clinical trials [103].

#### 3.2.2. Live Attenuated Whole-Cell Vaccines

Kim et al. reported the development of a live attenuated whole-cell vaccine that could provide serotype-independent protection. The *S. pneumoniae* D39 was attenuated by removal of the *pep27* gene (Δ*pep27*) and this led to the inability of bacteria to attach or colonize the lungs, blood, and brain [96]. The study also revealed that long-lasting protection against heterologous strains was observed in mice immunized with adjuvant-free intranasal Δ*pep27* pneumococcus [15]. Moreover, immunization with the Δ*pep27* pneumococcus was able to confer protection against secondary pneumococcal infections [104]. However, during immunization, there is a possibility of reversion of the Δ*pep27* pneumococcus to the wild type phenotype. To enhance the safety of the vaccine, *comD* was also deleted as it plays an important role in activating competence. The results showed that the reversion of the Δ*pep27* pneumococcus was eliminated by the removal of the additional *comD* gene. Immunization of the mice with the Δ*pep27*Δ*comD* double mutant significantly increased the IgG titers against pneumococcus serotype D39, which were 50-fold higher in IgG titers between the first and fourth immunizations. Immunization with the Δ*pep27*Δ*comD* mutant strain was found to elicit PspA-specific IgG. One week after the fourth weekly immunization, challenge of the mice against serotypes D39 or 6B showed a significant increase in the survival rate (>80%) when compared to the control group. The Δ*pep27*Δ*comD* strain immunization reduced the colonization levels regardless of the serotype and a non-typeable strain (NCC1). Moreover, the safety of the double mutant strain in normal and immunocompromised mice was confirmed, which indicated that the Δ*pep27*Δ*comD* strain could be used as a safe and effective vaccine against pneumococci [96].

Jang et al. also constructed a live attenuated vaccine strain by removing the prolipoprotein diacylglycerol transferase (*lgt*) gene from the capsule of the pneumococcal strain TIGR4 (TIGR4Δ*lgt*). The vaccine strain was able to exhibit protection against heterologous pneumococcal strains. Attenuation of the encapsulated pneumococcal strain TIGR4 by the removal of the *lgt* gene resulted in a reduction of virulence and inflammatory activities. A certain period of colonization of the TIGR4Δ*lgt* strain in the nasopharynx could induce strong mucosal IgA and IgG2b-dominant systemic antibody responses, which showed cross-reactivity to different pneumococcal serotypes. In addition, intranasal immunization of mice with TIGR4Δ*lgt* conferred protection against pneumococcal challenge with serotypes 2 (D39), 3(wu2), 6B, 9V, 19F, and 23F strains. The immunogenicity response showed that TIGR4Δ*lgt* could serve as an attractive broad-spectrum pneumococcal vaccine candidate [105]. *S. pneumoniae* serotype 1 is the dominant pathogen associated with invasive disease in sub-Saharan Africa despite their low carriage in the healthy population [106]. Terra et al. constructed a serotype 1 strain 519/43 that carried a defined pneumolysin (Ply) mutation (519/43Δply), which caused a loss of its ability to lyse red blood cells. Upon intraperitoneal challenge of mice immunized with strain 519/43Δply, there were fewer bacteria present in the blood but there was no complete protection from pneumoniae. Thus, Ply in strain 519/43 might be a weak virulence determinant [107]. This is in contrast to its presence in the killed whole-cell vaccine strain R200, which had its *Ply* gene being replaced with pneumolysoid, which carried three mutations in the *Ply* gene [101].

## 4. Promising Pneumococcal Vaccines in Clinical Trials

There are a number of pneumococcal candidate vaccines under development. A comprehensive list of the vaccine candidates and those in clinical trials were reviewed by Lagousi et al., Pichichero and Kim et al. [55,57,108]. In this review, we will only discuss those that appear to have progressed substantially and the potential to reach the market.

Merck Sharp & Dohme Corp. (Kenilworth, NJ, USA) is currently conducting Phase 3 clinical trials investigating a new conjugate vaccine, PCV-15 (V114), in healthy infants (42 to 90 days) (ClinicalTrials.gov Identifier: NCT03692871) and in healthy adults 50 years of age and above (ClinicalTrials.gov Identifier: NCT03480763) as well as in adults 50 years of age and above with increased risks (ClinicalTrials.gov Identifier: NCT03547167). The PCV-15 (V114) included the 13-valent PCV-13 plus serotypes 22F and 33F [109]. In previous Phase 1 and 2 studies, PCV15 displayed acceptable safety profiles and induced IgG and opsonophagocytic activity to all 15 vaccine serotypes at levels comparable to PCV13 for 10 of the 13 shared serotypes in healthy infants (≥42 days to ≤89 days) [109] and adults ≥50 years of age [110]. 

Pfizer Inc. (New York, NY, USA) evaluated the 20-valent pneumococcal conjugate vaccine (20vPnC). The 20vPnC vaccine includes the 13 serotypes from Prevnar 13 plus seven additional serotypes (8, 10A, 11A, 12F, 15B, 22F, and 33F) which are known to cause IPD [21,111,112,113,114] and meningitis [115,116]. The seven new serotypes are often associated with antibiotic resistance [114,117,118] and high fatality [119,120,121,122]. The phase 1 clinical trial in healthy adults (18–49 years) induced substantial functional (OPA) and IgG responses to all vaccine serotypes [123]. The promising Phase 2 clinical trial data have progressed the 20vPnC vaccine to Phase 3. The Phase 3 clinical trials (NCT03828617, NCT03835975, and NCT03760146) have been conducted in 6000 adults, which include populations of vaccine-naïve adults and adults with prior pneumococcal vaccination. The safety and immunogenicity findings were assayed by measuring antibodies associated with serotype-specific bacterial killing [opsonophagocytic activity (OPA)] prior to vaccination [124].

*S. pneumoniae* killed whole-cell vaccine (WCV) has the capacity to confer broader protection against *S. pneumoniae* as many of the antigens were presented in their native conformations and would be exposed to the host immune system [102]. The WCV vaccine was manufactured at Walter Reed Army Institute of Research (USA) from strain RM200 (RX1E PdT *ΔlytA*) and inactivated with beta-propiolactone. The WCV vaccine was administered with aluminum hydroxide in healthy young adults in a 3-vaccination series in a Phase 1 trial. The vaccine showed favorable safety, tolerability, and immunogenicity profile [103]. A Phase 2 study was conducted by PATH (Seattle, WA, USA) to find the optimal dosage for WCV and was recently evaluated in a clinical trial in healthy young Kenyan adults (18 to 45 years) and toddlers (12 to 15 months) (ClinicalTrials.gov Identifier: NCT02097472).

A novel pneumococcal vaccine, PnuBioVax, was constructed by mutating the pneumococcal toxin, pneumolysin, of *S. pneumoniae* serotype 4 TIGR4 strain into a non-toxic form. The mutation was constructed such that the immunogenicity of the toxin was preserved and acted as a potent activator of the complement system [125], the toll-like receptor 4 [81] and CD4 T cell migration [126]. The genetically-modified bacteria were then subjected to heat stress, induced by a temperature shift from 30 °C to 37 °C to mimic the translocation of the bacteria from being a commensal in the nasopharynx to a pathogen in sterile sites such as the lungs and blood. In theory, this process would upregulate and enrich for the surface proteins (PspA, PsaA, PiuA and non-toxic pneumolysin) associated with disease manifestations. This cocktail of proteins constituted the multi-antigen protein vaccine. In the preclinical study, rabbit sera immunized with PnuBioVax showed the killing of the vaccine strainTIGR4, and also serotype strains 6B, 19F, and 15B in an opsonophagocytic killing assay. Moreover, incubation of a number of pneumococcal strains in the immunized sera led to agglutination of the bacteria, inhibition of pneumolysin-mediated lysis of erythrocytes and reduced bacterial invasion of lung epithelial cells in vitro [127,128]. Hence, PnuBioVax offers the potential for broad-based protection via multiple mechanisms of action irrespective of serotypes. PnuBioVax had undergone a Phase 1 clinical trial (ClinicalTrials.gov Identifier: NCT02572635) sponsored by ImmunoBiology Limited (UK) and it was found to be safe and immunogenic in healthy adults (18-40 years) [128]. 

The protein-based vaccine, PPrV, is a trivalent protein vaccine carrying combined recombinant proteins, PcpA, PhtD, and PlyD1. Previously, it was shown that vaccination with PPrV protected mice against lethal pneumonia in an infant murine model [129]. Following that, a Phase 1 study was conducted by Sanofi Pasteur (France) and PPrV was found to be safe and immunogenic in adults, toddlers and infants [130]. The study has since proceeded to Phase 2 trial, which investigated the vaccine reactogenicity, safety, and immunogenicity when co-administered with the existing PHiD-CV vaccine. This form of combination therapy might also provide a broader range of protection against pneumococcal diseases [131]. Vaccination with PHiD-CV/dPly/PhtD-30 in pneumococcal vaccine-naïve children was well-tolerated and immunogenic [132]. PPrV did confer protection by macrophage- and complement-dependent mechanisms [133] as well as by reducing pneumococcal adherence and nasopharyngeal colonization [134].

Three recombinant avirulent PspA-protein based vaccines were constructed by researchers from Arizona State University [135]. Parenteral immunization with PspA has been shown to be protective in a lethal challenge study [136,137]. An oral route of vaccine administration for the pneumococcal vaccine has also been explored. In a Phase 1 clinical study, the three recombinant avirulent *Salmonella Typhi* (RASV) strains, each expressing PspA, were compared as live vaccines to evaluate the safety and tolerability of a single oral dosage in adult subjects (ClinicalTrials.gov Identifier: NCT01033409). Effective protection against secondary pneumococcal pneumonia was previously achieved by oral vaccination with an attenuated *Salmonella* delivering the PspA antigen in mice [138]. Despite PspA being known to be serologically variable, a common major histocompatibility complex (MHC-II) epitope against PspA was identified based on in silico prediction and ex vivo screening. The epitope was shown to be necessary for inducing interleukin-17 A (IL-17A) and it was associated with cross-protection [139].

Pneumolysin (Ply) and pneumococcal histidine triad protein D (PhtD), either administered alone or in combination, were shown to protect the mice against lethal challenges, pneumococcal disease and nasopharyngeal carriage (NPC) [86,140]. Clinical trials were conducted by GSK (Belgium) to evaluate the safety, reactogenicity, and immunogenicity of different pneumococcal protein-based formulations in adults, toddlers, and infants. In a Phase 1 study (Clinicaltrials.gov Identifier: NCT00707798), healthy adults (18–40 years) received two doses of one of six different formulations containing pneumolysin toxoid (dPly) or PhtD alone or a mixture of the two proteins (dPly and PhtD) or a combination of dPly and PhtD with the 10-valent pneumococcal non-typeable *Haemophilus influenza* protein D conjugate vaccine (PHiD-CV). The control group received a single dose of the 23-valent pneumococcal polysaccharide vaccine (23PPV; Pneumovax23TM, Sanofi Pasteur MSD). The results showed that the vaccine formulations containing dPly and PhtD, either alone or combined with PHiD-CV, were well tolerated and immunogenic [141]. In step one of phase 2, (Clinicaltrials.gov Identifier: NCT00985751), toddlers (12–23 months) received one of four investigational vaccine formulations containing a mixture of dPly and PhtD or in combination with PHiD-CV. Licensed PHiD-CV was used as a control [142]. All the vaccine formulations were well-tolerated and immunogenic upon administration as a two-dose primary vaccination followed by a booster dose. In step two of Phase 2 (ClinicalTrials.gov identifier: NCT01262872), the impact of two formulations of vaccines containing dPly and PhtD combined with PHiD-CV were evaluated in infants (aged 8-10 weeks). The inclusion of pneumococcal proteins in the PHiD-CV/ dPly/PhtD vaccine showed no impact on pneumococcal NPC prevalence, despite the success of these vaccines in preclinical trials in adults and toddlers. This could be due to the different immunization routes, differences in the adjuvant and/or initial immune status of mice versus infants. However, the quantitative relationship between the impact of pneumococcal protein-based vaccines on NPC prevalence versus pneumococcal disease in humans is yet to be defined, and no effect on carriage does not preclude protection against disease [143].

## 5. Development of Pneumococcal Nanovaccines

Vaccines are commonly administered via intramuscular injection routes but they are poor inducers of mucosal immunity through this route. Furthermore, antigens are usually poorly immunogenic and they require a suitable adjuvant or delivery system to deliver them through the oral, nasal, or mucosal routes in order to facilitate the enhancement of antigen uptake and prolong antigen availability. Thus, the use of adjuvants and delivery vehicles are important for the development of effective vaccines. The current microparticles and nanoparticles that have been used in *Pneumococcal* antigen delivery are listed in Table 2.

Protein-based vaccine such as PspA is a promising candidate for the development of a universal serotype-independent vaccine. The entrapment of the PspA antigen in polymeric particles such as poly-lactic acid (PLA) and poly (glycerol adipate-co-ω-pentadecalactone) (PGA-co-PDL) was stable and found to elicit robust IgG responses in mice and rats. The protein integrity and antigenicity of PspA were reported to remain unchanged [144,145].

Rodrigues et al. developed PGA-co-PDL polymeric nanoparticles (NPs) adsorbed with PspA from clade 4 of family 2 (PspA4Pro) within L-leucine microcarriers (nanocomposite microparticles-NCMPs) for mucosal delivery targeting the lungs (NP/NCMP PspA4Pro). The anti-PspA4Pro IgG antibodies were induced in serum and lungs of the immunized mice. Serum IgG exhibited good binding efficiency towards intact bacteria that expressed PspA from clades 3, 4, and 5 of pneumococcus family 2. However, no binding to bacteria expressing PspA from clades 1 and 2 of family 1 was detected. In addition, immunizing the mucus membrane with NP/NCMP PspA4Pro was capable of inducing local and systemic antibodies that conferred partial protection against pneumococcus family 2 alone [146].

Bacterium-like particles (BLPs) have the potential to serve as an immune stimulant in mucosal vaccine applications. Wang et al. developed a novel PspA protein vaccine based on BLP delivery system that could provide broad protection against pneumococcal pneumonia in mice [147]. The shape and size of BLPs are similar to bacteria (between 0.5 to 5μm) and were made from the food-grade bacterium *Lactococcus lactis* through simple hot acid treatment. BLPs could serve as a carrier for PspA protein bound to their surface through interaction with the protein anchor (PA) of *L. lactis*. BLPs could activate antigen-presenting cells and act as a potent immune stimulant for mucosal immunization [148]. Lu et al. combined PspA from families 1 and 2 into BLP-based pneumococcal vaccines so as to confer broader protection [149]. They successfully showed induction of high levels of serum IgG and mucosal specific IgA against challenge in the murine model. Immunization with the PspA-BLP vaccine protected against lethal intranasal challenge with pneumococci from families 1 and 2 regardless of the serotype [147,149].

Chitosan is a non-toxic, biodegradable, biocompatible natural polysaccharide and it was approved by the Food and Drug Administration (FDA) as a safe adjuvant and vaccine delivery system [150,151]. In addition, the chitosan formulation was able to improve the immunogenicity of a peptide-based vaccine and induced an immune response mediated by IgG2a [152]. Chitosan-DNA nanoparticles expressing PsaA pneumococcal vaccine was able to induce mucosal and systemic immune responses as well as increasing the protection against pneumococcal nasopharyngeal colonization [153]. Furthermore, Xu et al. used chitosan to deliver the PsaA protein vaccine to immunize BALB/c mice intranasally. Levels of IFN-γ, IL-17A, and IL-4 as well as IgG and mucosal IgA were significantly enhanced. In an intraperitoneal challenge with pneumococcus serotype 3 or serotype 14, the survival rate of mice immunized with chitosan–PsaA nanoparticles was improved [154]. In another study by Haryono et al., the chitosan nanoparticles prepared by ionic gelation and dry method were mixed with the 13-valent pneumococcal conjugate vaccine (PCV13; Prevnar^®^; Pfizer Inc., New York, NY, USA) as a PCV antigen model. The mice immunized with the PCV co-delivered with chitosan nanoparticles were found to significantly enhanced anti- Pn14PS (pneumococcal type 14 polysaccharide) IgG subclass antibodies such as IgG1, IgG2a, IgG2b, and IgG3. The antibodies were at higher levels than when the Quil-A adjuvant was used as a control co-delivery system [155].

Tada et al. showed that the cationic liposomes (DOTAP/DC-chol liposomes) were able to enhance the delivery of PspA to nasal dendritic cells of mice and conferred complete protection against lethal infection *S. pneumoniae*. The DOTAP/DC-chol liposomes could be a potential adjuvant for nasal delivery of pneumococcal vaccines as liposomes are biocompatible and biodegradable in the human body. In general, size, surface charge, and chemical composition of liposomes are important factors to be taken into consideration in the development of liposomes as the delivery system. However, limited studies of liposomes as an adjuvant in pneumococcal vaccine development have been published [156].

Nanoparticles have been shown to be efficient in vaccine delivery systems. Gold nanoparticles (GNPs) are small with a diameter of 1 to 100 nm. Gold nanoparticles carrying 45% of tetrasaccharide derived from the *S. pneumonia* type 14 capsular polysaccharide (Pn14PS) and 5% ovalbumin 323–339 peptide (OVA_323–339_) were able to elicit antibodies that promoted the phagocytosis of the pneumococcus. Additionally, T-helper cell activation was promoted [157,158].

Polyanhydrides are nanoparticles that were formulated using random copolymers based on 1,8-bis-(p-carboxyphenoxy)-3,6-dioxaoctane (CPTEG), 1,6-bis-(p-carboxyphenoxy) hexane (CPH), and sebacic acid (SA) which were able to show beneficial vaccine delivery and adjuvant properties. In addition, hydrophobicity, and surface erosion characteristics of polyanhydrides helped to stabilize labile proteins and protected them from denaturation by enzymatic cleavage and acidic degradation. Polyanhydride nanoparticles when administrated as a single dose vaccine was able to prolong the release of antigens that continued to drive the adaptive immune response [159,160]. Wagner-Muniz et al. designed a PspA-based polyanhydride nanovaccine by fully encapsulating the protein within the polyanhydride nanoparticles. The vaccine was stable at room temperature and immunization of mice with a single dose of the nanovaccine presented significantly higher survival rates (87.5%), even with a 25-fold reduction in the antigen dose when challenged against *S. pneumoniae* (strain A66.1 clad 2 of family 1). This comparison was in contrast to the control mice which were immunized with the soluble PspA protein alone. The efficacy of the nanovaccine was stable even after dry storage for 60 days at room temperature [161].

Lipoproteins are highly conserved among *S. pneumoniae* and are highly immunogenic. Hence, they can serve as a useful target for future vaccine development. Voß et al. immunized mice intranasally with the recombinant proteins, pneumococcal nucleoside-binding protein (PnrA), methionine-binding protein (MetQ), L,D-carboxypeptidase (DacB), and PsaA, using cholera toxin subunit B (CTB) as an adjuvant. Following the intranasal challenge with *S. pneumoniae* D39, the PnrA lipoprotein vaccine was able to prevent nasopharyngeal colonization. The protective effects of MetQ and DacB were insignificant [162].

Meanwhile, nanogel, a safe and effective nanosize nasal vaccine delivery system based on cationic cholesteryl group-bearing pullulan (cCHP) containing PspA was developed by Fukuyama et al. [163]. The colonization and invasion by pneumococcus in both the upper and lower respiratory tracts were reduced and high levels of systemic and nasal mucosal Th17 responses were induced in the infected airway of the cynomolgus macaques (*Macaca fascicularis*) model. The cCHP nanogel could potentially be employed as an antigen-delivery carrier for adjuvant-free nasal vaccination for the prevention of pneumonia in humans [69,163].

## 6. Conclusions

*Streptococcus pneumoniae* is responsible for non-invasive diseases such as acute otitis media and sinusitis in the community, but infection can also lead to serious complications, such as meningitis and septicemia. Mortality rates are high especially in the very young, elderly, and immunocompromised individuals. Despite the success of the polysaccharide PPV23 and the conjugated PCV10/13 vaccines to reduce pneumococcal infections, several issues remain, such as inadequate protection against capsular serotypes, which are not included in the vaccine and the unencapsulated *Streptococcus pneumoniae* (NESp). PPV23 has low immunogenicity and generates low immune memory response in the very young. New PCV-based vaccines would require country- and serotype-specific antigens, which could be changing continuously and placing limitations in making new PCV-based vaccines. The live-attenuated whole-cell vaccine posed concern about reversion to pathogenicity, while the inactivated whole-cell vaccine required multiple dosages and produced non-lasting humoral responses.

This study highlights the new approaches to design an effective pneumococcal vaccine. A live-attenuated vaccine with low pathogenicity could be constructed by introducing multiple mutations as well as deletions in the pneumococcal genome. For example, a mutant deficient in *pep27* and *comD* genes with reduced transformation ability could be further mutated in virulence genes, such as *PspA*, *PsaA*, *PspC*, and pneumolysin (*Ply*). The protein-based vaccines in early development were based on the full-length protein, which had high sequence variability and differences in levels of expression in different serotypes. The identification of highly conserved antigenic regions within the virulence proteins enables the use of distinct protein fragments. This allows the construction of combined antigenic regions represented by peptides. Due to the low immunogenicity of peptides, they could be conjugated to Toll-like receptors (TLRs) or nanoparticles. Further design of protein-based vaccines conferring serotype independent protection might be achieved through the development of bacterium-like particles (BLPs) or microparticles/nanoparticles presenting multiple virulence surface proteins such as PspA2 in combination with PspA4 and PsaA. BLPs displaying multiple antigens or nanoparticles formulated with pneumococcal surface proteins looked like promising strategies for mucosal immunization to enhance both systemic and mucosal immune responses.

## Figures and Tables

**Table 1:** Localization and function of *Streptococcus pneumoniae* virulence proteins that are well conserved among different serotypes.

Protein	Localization	Function	References
Pneumococcal surface protein A (PspA)	Surface protein	Inhibits complement-mediated clearance of pneumococci	[60,61,62]
Pneumococcal surface protein C (PspC)	Surface protein	Adhesin, interacts with human secretory immunoglobulin A (IgA) and immunoglobulin receptor (pIgR), inhibits complement activation by interacting components C3 and factor H (FH) to inhibit pneumococcus binding	[63,64]
Pneumolysin (Ply)	Cytoplasm/Cell wall	Able to insert into eukaryotic cell membranes and oligomerize to form large transmembrane pores which lead to cell lysis	[65,66]
Pneumococcal histidine triad protein D (PhtD)	Surface protein	Adhesin, high affinity to bind to Zinc ion	[67]
Elongation factor Tu (EF-Tu)	Surface protein	Catalyzes the binding of an aminoacyl-tRNA (aa-tRNA) to the ribosome. Inhibit protein synthesis	[68]
Pneumococcal peptide 27 (Pep27)	Cell membrane	Enable the bacteria to attach or colonize in the lungs, blood, and brain	[15]

**Table 2:** Microparticles and nanoparticles for *Pneumococcal* antigen delivery.

Nanoparticles	Characteristics of Nanoparticles	Pneumococcal Strains Used in Challenge Studies	Antigen (s)	Results	References
Polylactide (PLA)	45 kDa, 2–8 μm	n/a	PspA	The entrapment of PspA in PLA particles was observed to be stable. Polymer particles entrapping PspA elicited robust IgG responses in both mice and in rats. Protein integrity of antigen load in microparticles was maintained.	[144]
Poly(glycerol adipate-co-ωpentadecalactone) (PGA-co-PDL)	322.83 ± 4.25 nm (with the adsorption of PspA4Pro)	n/a	PspA4Pro (PspA clade 4)	Antigen PspA4Pro release from PGA-co-PDL nanoparticle was confirmed. PspA4Pro antigenicity was retained and the functional epitopes of the antigen were active in the formulation.	[145]
Nanocomposite microparticles (NP/NCMP)	~2 μm	EF3030 (serotype 19F, PspA1) ATCC6303 (serotype 3, PspA5)	PspA4Pro (PspA clade 4, encompassing mature N-terminal region till proline-rich region)	Anti-PspA4Pro IgG antibodies in serum and lungs were induced. Binding analysis of serum IgG to intact bacteria revealed efficient binding to bacteria expressing PspA from clades 3, 4, and 5 (family 2). However, no binding to bacteria expressing PspA from clades 1 and 2 (family 1) was observed. Immunization with NP/NCMP PspA4Pro was unable to decrease the bacterial load in the lungs following challenge with a serotype 19F strain expressing PspA from clade 1 (PspA1). Local and systemic antibodies were induced and offered protection against only one strain expressing PspA in homologous family 2.	[146]
Bacterium-like particles (BLP)	Similar to the size of the bacterium—0.5 to 5μm	ATCC6303 (PspA family 2 clade 5, serotype 3) ATCC101813 (PspA family 1 clade 2, serotype 3)	PspA, family2 clade-4 (derived from N-terminal α-helical region and the proline-rich region of pneumococcal strain EF5668	Safe and affordable. Both PspA-specific IgG and PspA-specific IgA were induced. Complete protection in a mouse challenge model (immunized with BLPs/PspA-PA) with pneumococci from two different clades of both homologous and heterologous PspA families that led to high protection against challenges with heterologous pneumococci and showed broader specificity.	[147]
		ATCC6304 (PspA family 1, clade 1) ATCC10813 (PspA family 1, clade 2) ATCC6319 (PspA family 2, clade 3) ATCC6314 (PspA family 2, clade 4) ATCC6303 (PspA family 2, clade 5)	PspA2 and PspA4	High levels of serum IgG and mucosal SIgA were induced. Binding to pneumococcal strains expressing PspA from clades 1 to 5 was observed. Immunization with the PspA-BLP vaccine conferred protection against fatal intranasal challenge with both PspA family 1 and family 2 pneumococcal strains.	[149]
Chitosan-DNA Nanoparticles	The average size of particle was 392 nm; zeta potential was +12.5 mV	*S. pneumoniae* ATCC 6303 (serotype 3)	pVAX1-PsaA (867-bp PsaA gene was amplified from *S. pneumoniae* genomic DNA ATCC 6303; serotype 3)	Anti-PsaA IgG antibody in serum and anti-IgA antibody in mucosal lavages were increased. Cellular immune responses were induced. Fewer pneumococci were recovered from the nasopharynx of mice immunized with chitosan-PsaA. Mucosal and systemic immune responses were generated and ultimately prevented pneumococcal nasopharyngeal colonization.	[153]
Chitosan-PsaA protein	The average size of particle was 691 nm; zeta potential was +21.1 mV	*S. pneumoniae* ATCC 6303 (serotype 3)	PsaA	The systemic (IgG in serum) and mucosal (IgA in mucosal lavage) specific antibodies were enhanced. Protection against acute otitis media following middle ear challenge with pneumococcus serotype 14 was increased The survival rate of mice immunized with chitosan–PsaA nanoparticles in an intraperitoneal challenge with pneumococcus serotype 3 or serotype 14 was improved.	[154]
Chitosan-PCV13	The particle size of 46.1nm		PCV13; Prevnar^®^; Pfizer Inc	No difference was observed in the level of total antibodies against Pn14PS antigen in the mouse groups with or without adjuvant codelivery. Both chitosan-PCV codelivery and Quil-A adjuvant (control) elicited IgG1, IgG2a, IgG2b, and IgG3 antibodies.	[155]
Cationic liposomes (DOTAP and DC-chol)	The particle size of 137.9 ± 11.6 nm; Zeta potential of 4.0 ± 2.1 mV	n/a	PspA, family 1 clade-2	Liposomes composed of 1,2-dioleoyl-3-trimethylammonium-propane (DOTAP) and cholesteryl 3β-N- (dimethylaminoethyl)carbamate (DC-chol) (DOTAP/DC-chol liposome). Provideed protective immunity against lethal inhalation of *S. pneumoniae*, and improved the survival rate of infected mice. Intranasal immunization with DOTAP/DC-chol liposomes carrying PspA induced both mucosal and systemic response. PspA-specific Th17 response was elicited. It plays a pivotal role in controlling *S. pneumoniae* infection by host innate immune response.	[156]
Gold nanoparticles (GNPs)	Mean gold core of 1.8 ± 0.5 nm; Average molecular weight of 84 to 97 kDa.	n/a	*S. pneumoniae* type 14, polysaccharide (Pn14PS) conjugated to CRM197 (Pn14PSCRM197), and T-helper ovalbumin 323–339 peptide (OVA_323–339_)	Specific anti-Pn14PS IgG antibodies were triggered. T-helper cell activation was promoted by glyconanoparticles The functionality of the antibodies was maintained as the antisaccharide antibodies promoted the phagocytosis of type 14 bacteria by human leukocytes.	[157]
Gold-glyco- nanoparticles	Mean gold core of 1.2 ± 0.3 nm; Average molecular weight of 45 to 55 kDa.	n/a	Serotype 14 (Tetra-14) and serotype 19F (Tri-19F), and a T-helper ovalbumin 323–339 peptide (OVAp)	A synthetic carbohydrate vaccine. GNPs functionalized with Pn19F and/or Pn14 saccharide ligands. The titers of specific IgG antibodies towards type 14 polysaccharide were enhanced.	[158]
PspA-based polyanhydride	The particle size of 50:50 CPTEG: CPH 455 ± 175; Zeta potential of −33.1 ± 5.1 mV	*S. pneumoniae* strain A66.1(PspA family 1, clade 2)	Pneumococcal surface protein A (PspA)	A room temperature stable PspA-based polyanhydride nanovaccine reduced the cost of vaccine care and transportation. Single immunization of the mice with nanovaccine and upon challenge presented significantly higher survival rates compared to mice immunized with soluble protein alone. A 25-fold reduction in protein dose of nanovaccine presented higher survival rates in immunized mice compared to the PspA soluble protein. A single dose of PspA-based nanovaccine against *S. pneumoniae* induced protective immunity.	[161]
Lipoprotein	n/a	*S. pneumoniae* D39	MetQ, PnrA, PspA and DacB proteins	High antibody titers were present in sera from mice immunized with the lipoproteins MetQ, PnrA, PsaA, and DacB.Mice were immunized intranasally with PnrA, DacB, and MetQ using cholera toxin subunit B (CTB) as an adjuvant, followed by an intranasal challenge with *S. pneumoniae* D39. No pneumococcal colonization was observed in vaccination using PnrA while DacB and MetQ led to reduction of the bacterial load. The increased production of antigen-specific IL-17A in the nasal cavity reduced bacteria colonization. High systemic IgG levels were induced with a predominance for the IgG1 isotype, except for DacB.	[162]
	~ 40 nm	*S. pneumoniae* Rx1	PspA, family 1, clades 2	cCHP nanogel contained 20 amino groups per 100 glucose units. Longer retention of PspA (in nanogel) was observed in the nasal cavity when compared with the administration of PspA alone. No deposition of [^18^F]-PspA was seen in the olfactory bulbs or brain. PspA-specific serum IgG with protective activity and mucosal secretory IgA (SIgA) Ab responses in cynomolgus macaques (*Macaca fascicularis*) were induced.	[163]

**Note:** n/a: not available; PspA: pneumococcal surface protein A; MetQ: methionine binding protein; PnrA: pneumococcal nucleoside-binding protein; DacB: L,D-carboxypeptidase; PsaA: pneumococcal surface adhesin A; cCHP: cationic cholesteryl group-bearing pullulan.
